# Re: Pegvaliase Treatment for Adolescents With Phenylketonuria: A Multi‐Site Study

**DOI:** 10.1002/jmd2.70085

**Published:** 2026-06-14

**Authors:** Patricia L. Hall, Coleman Turgeon, Dietrich Matern

**Affiliations:** ^1^ Biochemical Genetics Laboratory, Department of Laboratory Medicine and Pathology Mayo Clinic Rochester Minnesota USA

Dear Editors,

1

In the recent paper “Pegavaliase Treatment for Adolescents With Phenylketonuria: A Multi‐Site Study,” Hollander et al. use “blood” phenylalanine (Phe) levels as indicator of pegvaliase response [1]. While that is an obvious choice, we believe that any report that associates Phe concentrations to pegvaliase treatment should indicate the blood collection procedure, temperature during transport to the laboratory, and specimen type analyzed (i.e., plasma or dried blood spots). The reason is that the accuracy of Phe measurements in patients taking pegvaliase is dependent on all these factors. As we reported previously Phe can decrease in a blood specimen prior to analysis when the patient is treated with pegvaliase [2]. In plasma specimens, even during the short time needed to prepare a sample at room temperature for analysis, pegvaliase present in the specimen still degrades Phe resulting in a discrepancy of the in vitro versus in vivo concentration. The study's Phe decrease (50% reduction compared to before treatment was initiated) described to indicate achievement of treatment efficacy could easily be mimicked by routine sample handling at room temperature. The lowest Phe values observed (range 7–1418 μmol/L) in this study may be evidence of this phenomenon, as opposed to true Phe deficiency under treatment. If blood measurements are used as guide points to liberalize intact protein intake, understanding these preanalytical factors is key.

Of course, if the study adhered to the manufacturer's clinical trial manual, which recommends spinning down blood and freezing plasma within 30 min of collection, the reported Phe concentrations may be valid. But even under ideal conditions it is unlikely that a specimen will remain unfrozen for less than an hour when accounting for the above‐mentioned factors. It is likely that only a point‐of‐care Phe measurement could prevent these preanalytical effects, unless rigorous procedures were followed on the collection and the laboratory sides. From the laboratory perspective, such specimens would have to be marked to ensure the shortest processing time possible.

It is important to note that we do not believe that the lack of detailed blood collection and processing descriptions alters the overall findings and conclusions drawn from this study. However, those who treat patients with pegvaliase should be aware of the continued activity of pegvaliase in blood specimens after collection and consult with laboratories performing plasma amino acid analyses for their patients to ensure processes are in place to provide reliable Phe measurements. This is also important because sometimes specimens are exposed to longer times at room temperature due to unanticipated delays caused by assay failures or equipment access. Pegvaliase is an important new treatment option for patients with PKU; however, this medication has fundamental implications for accurate measurement and interpretation of Phe concentrations in blood.

## Funding

The authors have nothing to report.

## Conflicts of Interest

The authors declare no conflicts of interest.

## Data Availability

Data sharing not applicable to this article as no datasets were generated or analysed during the current study.
